# Anti-lysophosphatidic acid antibodies improve traumatic brain injury outcomes

**DOI:** 10.1186/1742-2094-11-37

**Published:** 2014-02-27

**Authors:** Peter J Crack, Moses Zhang, Maria Cristina Morganti-Kossmann, Andrew J Morris, Jonathan M Wojciak, Jonathan K Fleming, Ila Karve, David Wright, Maithili Sashindranath, Yona Goldshmit, Alison Conquest, Maria Daglas, Leigh A Johnston, Robert L Medcalf, Roger A Sabbadini, Alice Pébay

**Affiliations:** 1 Department of Pharmacology, the University of Melbourne, Parkville, Australia; 2 Department of Epidemiology and Preventive Medicine, Monash University, Melbourne, Australia; 3 Barrow Neurological Institute, Department of Child Health, Phoenix Children’s Hospital, University of Arizona, Phoenix, AZ, USA; 4 Division of Cardiovascular Medicine, University of Kentucky College of Medicine, Lexington, KY, USA; 5 Department of Biology, San Diego State University and Lpath Inc, 4025 Sorrento Valley Blvd, San Diego, CA, USA; 6 Florey Institute of Neuroscience and Mental Health, Parkville, Australia; 7 Department of Anatomy and Neuroscience, the University of Melbourne, Parkville, Australia; 8 Australian Centre for Blood Diseases, Monash University, Melbourne, Australia; 9 Centre for Eye Research Australia, Royal Victorian Eye and Ear Hospital & Department of Ophthalmology, the University of Melbourne, East Melbourne, Australia; 10 Australian Regenerative Medicine Institute, Monash University, Clayton, Australia; 11 National Trauma Research Institute, Alfred Hospital & Monash University, Melbourne, Australia; 12 Neuroengineering Laboratory, Department of Electrical and Electronic Engineering, the University of Melbourne, Parkville, Australia

**Keywords:** Lysophosphatidic acid, Traumatic brain injury, Human cerebrospinal fluid, Control cortical impact, Magnetic resonance imaging, Anti-LPA antibody, IL-6

## Abstract

**Background:**

Lysophosphatidic acid (LPA) is a bioactive phospholipid with a potentially causative role in neurotrauma. Blocking LPA signaling with the LPA-directed monoclonal antibody B3/Lpathomab is neuroprotective in the mouse spinal cord following injury.

**Findings:**

Here we investigated the use of this agent in treatment of secondary brain damage consequent to traumatic brain injury (TBI). LPA was elevated in cerebrospinal fluid (CSF) of patients with TBI compared to controls. LPA levels were also elevated in a mouse controlled cortical impact (CCI) model of TBI and B3 significantly reduced lesion volume by both histological and MRI assessments. Diminished tissue damage coincided with lower brain IL-6 levels and improvement in functional outcomes.

**Conclusions:**

This study presents a novel therapeutic approach for the treatment of TBI by blocking extracellular LPA signaling to minimize secondary brain damage and neurological dysfunction.

## Findings

Traumatic brain injury (TBI) is a major cause of brain neurotrauma, resulting in over 1.7 million brain injuries annually in the United States. TBI commonly results from motor vehicle accidents, falls, sports injuries or explosions, all of which disrupt normal brain function. The primary injury can be focal or diffuse and initially produces hemorrhage and axonal injury followed by secondary effects such as edema and ischemia arising from the activation of biochemical pathways including inflammation, cell death and gliosis [1]. These secondary-phase effects begin immediately to widen the area of injury and can persist for weeks to months, leading to profound behavioral deficits. Aggravated secondary brain damage is also thought to impair the ability of resident neuronal stem cells to regenerate the lost neurons. There are no FDA-approved drugs specific for TBI treatment and medical intervention is limited to supportive care. Thus, the ideal therapy for TBI would have the combined effects of reducing the initial injury as well as limiting the secondary inflammatory responses while simultaneously promoting regeneration and replacement of lost neural tissue.

Lysophosphatidic acid (LPA) is a bioactive, pro-inflammatory lysophospholipid found in the extracellular compartment, such as in blood and cerebrospinal fluid (CSF). LPA mainly acts through binding to its specific G-protein-coupled receptors LPA_1–7_ to induce pleiotropic effects on multiple cell types [2]. LPA is an inflammatory mediator, released by activated platelets, astrocytes and other pro-inflammatory cells. In the nervous system, although the role of LPA signaling in development has been described in detail, recent data also suggest an involvement of LPA in neurotrauma [2]. This is corroborated by compelling evidence from our group showing upregulation of LPA receptors following central nervous system (CNS) neurotrauma [3,4]. Using post-mortem human brains of normal individuals and patients who died following acute closed-head injury, we demonstrated that low constitutive expression levels of LPA receptors detected in normal human brain were upregulated following TBI. Interestingly, the upregulated expression of LPA_1_ co-localized with astrocytes while LPA_2_ was upregulated on the ependymal cell layer of the lateral ventricles [3]. Furthermore, using a mouse hemi-section model of spinal cord injury (SCI), we recently demonstrated that LPA is causally involved in the outcome of injury in that blocking its signaling via treatment with the specific anti-LPA monoclonal antibody (mAb), B3, limited glial scar formation, improved neuronal survival, promoted neurite sprouting and improved motor function [5]. We also observed that injection of LPA in vivo stimulated the inflammatory response and proliferation of glial cells in the Zebrafish following SCI [5]. In vitro studies also demonstrated the ability of the anti-LPA mAb to block receptor signaling in cells over-expressing LPA_1–3_ receptors [5].

Here, we report for the first time that LPA levels increase significantly in CSF samples taken from TBI patients as well as from mice subjected to control cortical impact (CCI) injury. Moreover, blocking LPA with the specific murine anti-LPA mAb, B3, improved neurological outcomes in the CCI mice. This study provides the first evidence that therapeutic anti-LPA mAbs could be useful in treating TBI-associated secondary damage.

## Methods

### Ethics

All experimental work performed in this study was approved by the Human or Animal Research Ethics committees of the University of Melbourne (HREC1136323, AEC0911437) or the Alfred Hospital (HREC194/05), in accordance with the requirements of the NHMRC.

### Animals

Eight-week-old male C57BL/6 J mice (23 ± 3 g) were intravenously administered, in a blinded fashion, a single dose of either murine IgG2β mAb targeted to LPA (B3, 25 mg/kg) [5] or an IgG2β isotype-matched mAb (25 mg/kg) either 1 h prior to, or 30 minutes post TBI. Injured mice were compared with uninjured sham controls. At all times, the investigator was blinded to the nature of the drugs to be delivered. Decoding of the samples was only performed once data were quantified.

### CSF measurement

Human CSF was obtained from patients with TBI admitted to the Alfred hospital with delayed informed consent obtained from the next of kin (Table 1). The inclusion criteria for the patients required that they had severe TBI with a post-resuscitation Glasgow Coma Scale (GCS) ≤8 (except one patient with GCS = 10) who rapidly deteriorated, requiring an extraventricular drain device (EVD) to monitor intracranial pressure (ICP) and therapeutic drainage of CSF [6]. Patient management included computed tomography (CT) scans within 4 h from TBI, followed by surgical implantation of an EVD. CSF was drained when the ICP exceeded 20 mmHg, and was collected daily beginning from the day of admission (day 0) up to day 5 after injury. Exclusion criteria were pregnancy, neurodegenerative diseases, HIV and other chronic infection/inflammatory diseases, or history of TBI. Clinical parameters were recorded by paramedics and medical staff, including GCS, hypotension (systolic blood pressure <90 mmHg) and occurrence of pre-hospital hypoxia. Patients were allocated to the hypoxic cohort (SaO_2_ < 92% or apnoeic or cyanotic at the field of the accident) or normoxic cohort (normal SaO_2_) [7]. TBI was classified into focal brain injury or diffuse brain injury following the Marshall CT score [8] as described [6]. The injury severity score (ISS) was assessed by including extracranial trauma [9]. The extended Glasgow outcome scale (GOSE) was assessed at 6 months post admission as described [6]. Control CSF samples were obtained from hydrocephalus patients undergoing implantation of ventriculo-peritoneal shunts with exclusion criteria similar to the TBI cohort.

**Table 1:** Demographic information for patients with severe traumatic brain injury

**Patient**	**Age**	**Sex**	**Oxygen saturation**	Injury*	**GCS**	**ISS**	**GOSE**
**1**	23	M	Normoxic	4	7	33	4
**2**	19	M	Normoxic	1	8	30	5
**3**	50	M	Hypoxic	1	5	41	4
**4**	33	M	Normoxic	4	4	38	1
**5**	40	M	Normoxic	6	7	21	6
**6**	33	M	Hypoxic	2	3	43	4
**7**	21	F	Normoxic	1	7	21	3
**8**	26	F	Hypoxic	6	3	45	3
**9**	22	M	Normoxic	5	7	30	5
**10**	35	M	Hypoxic	6	4	45	5
**11**	25	M	Hypoxic	1	4	41	N.A.
**12**	47	M	Normoxic	4	7	29	3
**13**	26	M	Normoxic	1	8	34	
**14**	29	F	Hypoxic	1	5	38	5
**15**	20	M	Normoxic	4	3	21	3
**16**	48	F	Hypoxic	8	3	34	1
**17**	22	M	Normoxic	1	8	34	
**18**	24	M	Hypoxic	1	3	34	5
**19**	19	M	N.A.	N.A.	N.A.	N.A.	N.A.
**20**	31	M	Hypoxic	1	5	43	2
**21**	39	M	Normoxic	6	7	26	8
**22**	25	F	Hypoxic	1	3	35	3
**23**	52	F	Normoxic	4	6	43	1
**24**	36	M	Normoxic	4	3	29	3
**25**	58	M	Normoxic	3	7	50	5
**26**	25	M	Normoxic	6	8	17	1
**Control 1**	42	M	Normoxic				
**Control 2**	80	M	Normoxic				
**Control 3**	56	M	Normoxic				

*Mechanism of iInjury: 1, motor vehicle accident; 2, motor bicycle accident; 3, pedal cyclist; 4. pedestrian; 5, penetrating injury; 6, fall/jump; 8, other; Glasgow Coma Scale (GCS) ≤8: severe; 9–12: moderate; ≥13: minor. ISS, injury severity score: 0, no injury; 75, maximal untreatable injury; GOSE, extended Glasgow outcome scale: 1, death; 2, vegetative state; 3, severe disability (lower band); 4, severe disability (upper band); 5, moderate disability (lower band); 6, moderate disability (upper band); 7, good recovery (lower band); 8, good recovery (upper band); M, male; F, female. N.A., not available.

### Liquid chromatography-mass spectrometry (LC-MS) methods

Levels of LPA were measured by high-performance liquid chromatography-electrospray ionization-tandem mass spectrometry as described previously [10]. Briefly, lipids were extracted from CSF samples using acidified organic solvents. Sample 10 to 50 mL was combined with 0.1 M HCL to a final volume of 0.5 mL in an 8-mL borosilicate glass tube containing 1 mL CHCl_3_ and 2 mL MeOH and 50 pmol of 1- hetpadecanoyl 2 hydroxy glycerol 3-phosphate (C17-LPA: Avanti Polar Lipids, Albaster, AL, USA). The tube was capped, vortexed and an additional 1 mL of CHCl_3_ and 1.3 mL 0.1 M HCl added. The tube was vortexed again and centrifuged (500 × g for 5 minutes) to separate the upper and lower phases. The lipid containing the lower phase was removed with a Pasteur pipette to a 4-mL borosilicate glass vial and evaporated to dryness under N_2_ gas using a Zymark Turbovap. The dried samples were resuspended in 0.1 mL MeOH and transferred to autosampler vials.

Lipids were analyzed using a Shimadzu UFLC coupled with an ABI 4000-Qtrap hybrid linear ion trap triple quadrupole mass spectrometer operated in multiple reaction monitoring (MRM) mode. Lipids were separated using an Agilent Zorbax Eclipse XDB C8 column, 5-μm, 4.6 × 150 mm column. The mobile phase consisted of 75/25 of methanol/water with formic acid (0.5%) and 5 mM ammonium formate (0.1%) as solvent A and 99/1 of methanol/water with formic acid (0.5%) and 5 mM ammonium formate (0.1%) as solvent B. Separation was achieved using a gradient of 0% B for 1 minute, 0% B to 100% B in the next 1 minute, maintained at 100% B for the next 10 minutes, and equilibrated to the initial conditions in 3 minutes. The flow rate was 0.5 mL/minute with a column temperature of 30°C. The sample injection volume was 10 μL. The mass spectrometer was operated in negative electrospray ionization mode with optimal ion source settings determined using a series of synthetic LPA species. The ion source settings were a declustering potential of 61 V, entrance potential of 10 V, collision energy of 23 V, collision cell exit potential of 16 V, curtain gas of 20 psi, ion spray voltage of 5,500 V, ion source gas1/gas2 of 40 psi, and temperature of 550°C. MRM transitions corresponding to 15 indicated abundant LPA molecular species and the C17 LPA internal standard were monitored. Quantitation was accomplished by correction for recovery of the C17 LPA internal standard with reference to calibration curves generated using authentic LPA standards (Avanti Polar Lipids) that were quantitated separately by phosphorous analysis after wet digestion in perchloric acid.

### Model of controlled cortical contusion in mice

Mice were subjected to CCI as described by Dixon et al. [11] in rats. Mice were anesthetized for 5 to 10 s with gaseous isoflurane (1 mL/minute), followed by an intra-peritoneal injection of ketamine (100 mg/kg, Parnell)/xylazine (10 mg/kg, Parnell). A sagittal scalp incision was made to expose the underlying parietal bone and a 2-mm burr hole was drilled using a Dremel 10.8 V drill with a 0.8 mm tip (Dremel, Europe) into the skull above the right parietal cortex, 1.5 mm posterior to the Bregma area and 2.5 mm lateral to the midline. The rounded section of bone was removed to expose the underlying cortex. Mice were placed on a stereotaxic frame and a 1.5-mm-deep impact was made into the brain on the exposed cortex using a computer-controlled impactor, with the center at coordinates anteroposterior = - 2.0, and mediolateral = + 2.0 from the Bregma area. The following parameters were set onto the program linked to the rounded tip impactor (LinMot-Talk 1100, Spreitenbach Switzerland): withdrawal of the tip 20 mm away from resting position at 1 m/s, impact at a velocity of 5 m/s and depth of 1.5 mm, interval of 100 ms and withdrawal of the tip 1.5 mm towards the resting position at 1 m/s. Following the impact, the removed bone section was placed back onto the skull, with a small amount of paraffin to close up the brain. The incision was closed up with a silk 5.0 metric suture (Syneture Tyco Healthcare, Dublin, Ireland). Mice were given Buprenorphine intra-peritoneally (0.6 mg/kg, Reckitt Benckiser Healthcare) and placed on a heat mat for post-surgical recovery. Sham controls underwent anesthesia, scalp incision and bone removal, but no injury, and then were sutured, given analgesic and put on a heat blanket for recovery.

### Mouse CSF sampling

CSF sampling from the cisterna magna of the mouse was carried out in a cohort of mice that had undergone TBI. Mice were anesthetised with an intraperitoneal injection of ketamine and xylazine and the head placed in a stereotaxic frame. After initial skin incision the subcutaneous tissue and muscles (*m. biventer cervicis* and *m. rectus capitis dorsalis major*) were separated by blunt dissection with forceps and a pair of microretractors was used to hold the muscles apart. Using a dissecting microscope the dura mater of the cisterna magna was punctured by a glass capillary tube and approximately 10 μL of CSF was extracted by capillary action and immediately frozen in liquid nitrogen for analysis.

### Behavioral analysis

Neurological function post TBI (at 48 h) was assessed using DigiGait™ v 11.5 (Mouse Specifics Inc., Quincy, MA, USA) apparatus as described in [12]. Mice were run on a transparent treadmill at a speed of 15 cm/s both before injury and post injury for 5 s. Videos of paw placement were captured in the ventral plane by the DigiGait™ software and analyzed by the software. All surgery and behavioral analyses for antibody-treated mice were performed in a blinded fashion. Gait measurements were calculated as post to pre-injury ratios of sham versus trauma mice. All gait parameters for antibody-treated mice were presented as fold change to trauma values. Statistical significance was determined by the Student *t*-test (unpaired, two-tailed) while comparing the raw data in each cohort, although the data have been depicted as fold-change relative to TBI.

### Magnetic resonance imaging (MRI)

Mice were intravenously injected with IgG isotype control (25 mg/kg) or LPA antibody (25 mg/kg) 30 minutes post TBI, and imaged both 1 day and 7 days after TBI. MRI scans were performed using a Bruker 4.7 Tesla small animal MRI scanner (Florey Institute of Neuroscience and Mental Health) to quantify the progression of tissue damage and the subsequent repair. Mice were initially anesthetized with approximately 3% isoflurane in a 1:1 mixture of medical-grade air and oxygen. Anesthesia was maintained throughout scanning with 0.25 to 1.5% isoflurane through a nosecone placed over the animal’s snout and respiration was continuously monitored throughout the experiment with a pressure-sensitive probe positioned under the animal’s diaphragm. Anesthetized animals were laid supine on a purpose-built small-animal holder and their heads fixed into position with ear and bite bars. A surface receiver coil was placed over the animals’ heads and the cradle was inserted into a transmitter coil fixed inside a BGA12S-HP gradient set for imaging. To accurately assess lesion size, groups of eight mice from each treatment were used. The MRI protocol consisted of a 3-plane localizer sequence followed by multi-echo T_2_ and diffusion-weighted sequences. The total scanning time was kept to less than 2 h per animal. Multi-echo T_2_-weighted images were acquired using a rapid acquisition, relaxation enhanced (RARE) sequence with RARE factor = 2; repetition time = 2,500 ms; effective echo time (TE_eff_) = 10, 30, 50, 70, 90 and 110 ms; field of view (FOV) = 1.6 × 1.6 cm^2^; matrix = 192 × 192; and 16 slices with thickness = 0.5 mm. The diffusion-weighted images were acquired with a diffusion-weighted echo-planar imaging (DWI-EPI) sequence with TR = 7.5 s; TE = 32.74 ms; repetitions = 3; FOV = 1.6 × 1.6 cm^2^; matrix = 64 × 64; 28 slices with thickness = 250 μm; 126 diffusion directions with diffusion gradient duration (δ) = 3 ms, diffusion gradient separation (Δ) = 14 ms and *b*-value = 1,200 s/mm^2^. Mean diffusivity maps were derived from the diffusion-weighted data. TBI-affected regions were manually outlined by a blinded operator on both T_2_ weighted and diffusion-weighted images using ImageJ software (NIH) and volumetric analysis carried out using Matlab (MathWorks, Natick, MA, USA).

### Histology and brain injury volume analysis

Brains were cut into 10-μm coronal sections starting at the rostral end, paraffin-embedded and mounted onto glass slides. Every tenth slide was stained with H&E. Photomicrographs were captured using a Zeiss Axioskop microscope and lesion area was determined using the Image J software (v1.47; NIH). Tissue swelling in the injured side was accounted for by dividing the lesion area from each section by the ratio of the areas of the injured relative to uninjuredside. The Cavalieri formula was used to calculate total lesion volume as follows:

Volume=ΣA×t×ISF

where A = sum of the corrected lesion areas; t = section thickness (10 μm) and ISF = inverse of the sampling fraction (1 in 10 sections was counted, that is, sampling fraction = 1/10). Lesion volume values were analyzed using the unpaired Student *t*-test, with a value of *P* <0.05 considered statistically significant.

### Cytokine measurements

ELISA kits (BD Biosciences, USA) were used to detect mIL-6 (kit # 555240), mTNF-α (kit # 560478) and mIL-1β (kit# 559603) levels in brain tissues as per manufacturer guidelines: 100 μg protein extract was loaded per well, with experiments conducted in duplicate. Protein concentrations of individual samples were determined using a linear standard curve of IL-6, TNF-α or IL-1β standards (4–200 pg/mL).

### Antibody-LPA binding measurements

B3 binding to individual LPA species was measured with the Kinetic Exclusion Assay (KinExA, Sapidyne Instruments, Boise, ID, USA) using a KinExA 3200 equipped with an autosampler. The LPA conjugate used to capture the free antibody was prepared by crosslinking 1-(12-mercaptododecanoyl)-2-hydroxy-/sn/-glycero-3-phosphate to maleimide-activated BSA (Thermo Scientific, Waltham, MA, USA) in 0.1 M sodium phosphate, 0.15 M NaCl, pH 7.2. The purified LPA-BSA conjugate was diluted with running buffer (PBS without calcium and magnesium (Cellgro, Manassas, VA, USA) with 0.002% azide), absorbed to PMMA beads (Sapidyne Instruments, Boise, ID, USA) and blocked with Fraction V fatty acid-free BSA (FAF-BSA, Calbiochem, USA). The 16:0, 18:0, 18:1, 20:4 acyl LPA species (Avanti Polar Lipids) and 18:2 acyl LPA (Echelon Bioscience, Salt Lake City, UT, USA) were weighed out in amber glass vials and dissolved in 100% methanol by repeated sonication and vortex mixing as needed until the solutions were clear. Aliquots (1 to 3 μmol) were transferred to new amber glass vials, and the methanol was evaporated using a dry argon stream. The dried LPA aliquots were resuspended in running buffer containing 1 mg/mL FAF-BSA by repeated sonication and vortex mixing to a final LPA concentration of 0.5 mM.

Samples containing 10 μM of each LPA species (100 μM 18:0 LPA), 1 nM B3 antibody and 3 μM FAF-BSA in the KinExA running buffer were prepared in silanized glass tubes. Using a glass syringe, 1 mL of each sample was transferred to a glass tube containing 2 mL of a receptor solution (1 nM B3, 3 μM FAF-BSA in running buffer) and gently mixed. This 3-fold serial dilution was repeated until 14 sample fractions were prepared for each LPA species. Sample fractions were equilibrated for >1 h at room temperature prior to performing equilibrium affinity experiments. B3 captured on the beads was detected using a DyLight sheep anti-mouse heavy and light chain secondary (Jackson ImmunoResearch, West Grove, PA, USA) at 375 ng/mL in running buffer. Each fraction was analyzed in duplicate using the KinExA Pro software version 3.6.3 (Sapidyne Instruments, Boise, ID, USA).

## Results

### LPA levels are elevated in human and mouse CSF following TBI

**Figure 1:**
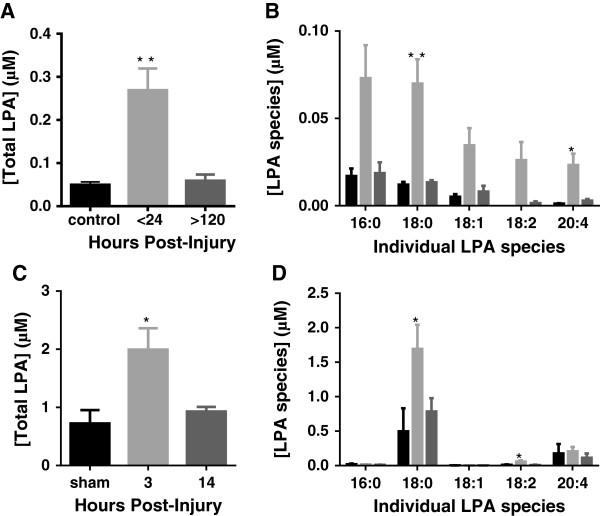
**Cerebrospinal fluid (CSF) analysis of lysophosphatidic acid (LPA) content following traumatic brain injury (TBI).** Total **(A)** and individual **(B)** LPA levels (means ± standard error of the mean (SEM)) in the CSF of patients with non-penetrating TBI at different time points (control, n = 3; <24 h, n = 18; >120 hours, n = 11). (F(2,29) = 6.453, *P* = 0.005 (one-way analysis of variance (ANOVA)); Tukey multiple comparisons tests: <24 h versus >120 h (Total), **P = 0.007; <24 h versus >120 h (18:0), **P = 0.008; <24 h versus >120 h (20:4), **P = 0.04. Total **(C)** and individual **(D)** LPA levels (means ± SEM) in the CSF of mice post control cortical impact (CCI) at different time points (sham, n = 4; 3 h, n = 6; 14 h, n = 6). (F(2,13) = 6.674, *P* = 0.01) by one-way ANOVA; Tukey multiple comparisons test: 3 h versus sham (Total), *P = 0.02 and 3 h versus 14 h (Total), *P = 0.03; 3 h versus sham (18:0), *P = 0.01 and 3 h versus 14 h (18:0), *P = 0.03; 3 h versus sham (18:2), *P = 0.03 and 3 h versus 14 h (18:2), *P = 0.01.

Our previous work showed that LPA receptors were upregulated following brain trauma in mice and humans [3,4]. Although these key components of the LPA signaling pathway were upregulated, the levels of LPA were not assessed after injury. Consequently, we report here for the first time, elevated levels of total LPA in human CSF obtained from patients with TBI compared to non-injured control individuals (Figure 1A-B). In TBI patients, levels of LPA in the CSF were substantially and significantly increased from 0.050 ± 0.007 μM in control samples to 0.270 ± 0.050 μM in the first 24 h and returned to basal levels by 120 h (0.059 ± 0.014 μM), showing that the LPA-pulse usually occurs within the first 24 h after injury (Figure 1A). Figure 1B depicts the distribution of LPA isoforms in the human CSF showing that 16:0 and 18:0 LPA are the predominant isoforms contributing to the total LPA pulse. These data thus suggest that LPA production and/or degradation in CSF may be dysregulated/upregulated early in the injury process.

Before testing the therapeutic value of our anti-LPA mAbs in a mouse model of TBI, we first sought to corroborate the human findings as to whether LPA levels were also upregulated in the CSF of injured mice. We employed the CCI model of TBI as this closely reproduces the closed TBI of our patient population [13]. As shown in Figure 1C, total LPA levels increased in the CSF of CCI-injured mice 3 h post TBI, returning to baseline levels at 14 h post injury. A comparison of Figures 1A versus 1C reinforces the similarity of our findings between the CCI model and human TBI, as both displayed dysregulation of LPA in the early hours post injury. We also performed lipidomics analysis to determine the isoform distribution of LPA species in the CSF after injury. Interestingly, Figure 1D shows that 18:0 LPA is by far the predominant LPA species contributing to the LPA-pulse produced by the injured mouse brain.

### Anti-LPA mAbs reduce the lesion size following TBI

Having demonstrated the upregulation of the LPA target in the CCI model (Figure 1C-D), it was important to demonstrate a causal connection between elevated LPA levels in the brain and the progression of secondary tissue injury. Accordingly, we assessed the ability of a specific anti-LPA mAb (B3/Lpathomab), to block the pathological actions of LPA in CCI mice. We had previously shown potency of this antibody in blocking LPA receptor signaling as well as the specificity of this antibody towards LPA [5]. We adapted a CCI model originally described in the rat, which shows a high survival rate but no chronic neurological impairment [11]. In our model of CCI, the impactor only travels to 1.5 mm of depth within the brain, resulting in a mild cortical lesion.

**Figure 2:**
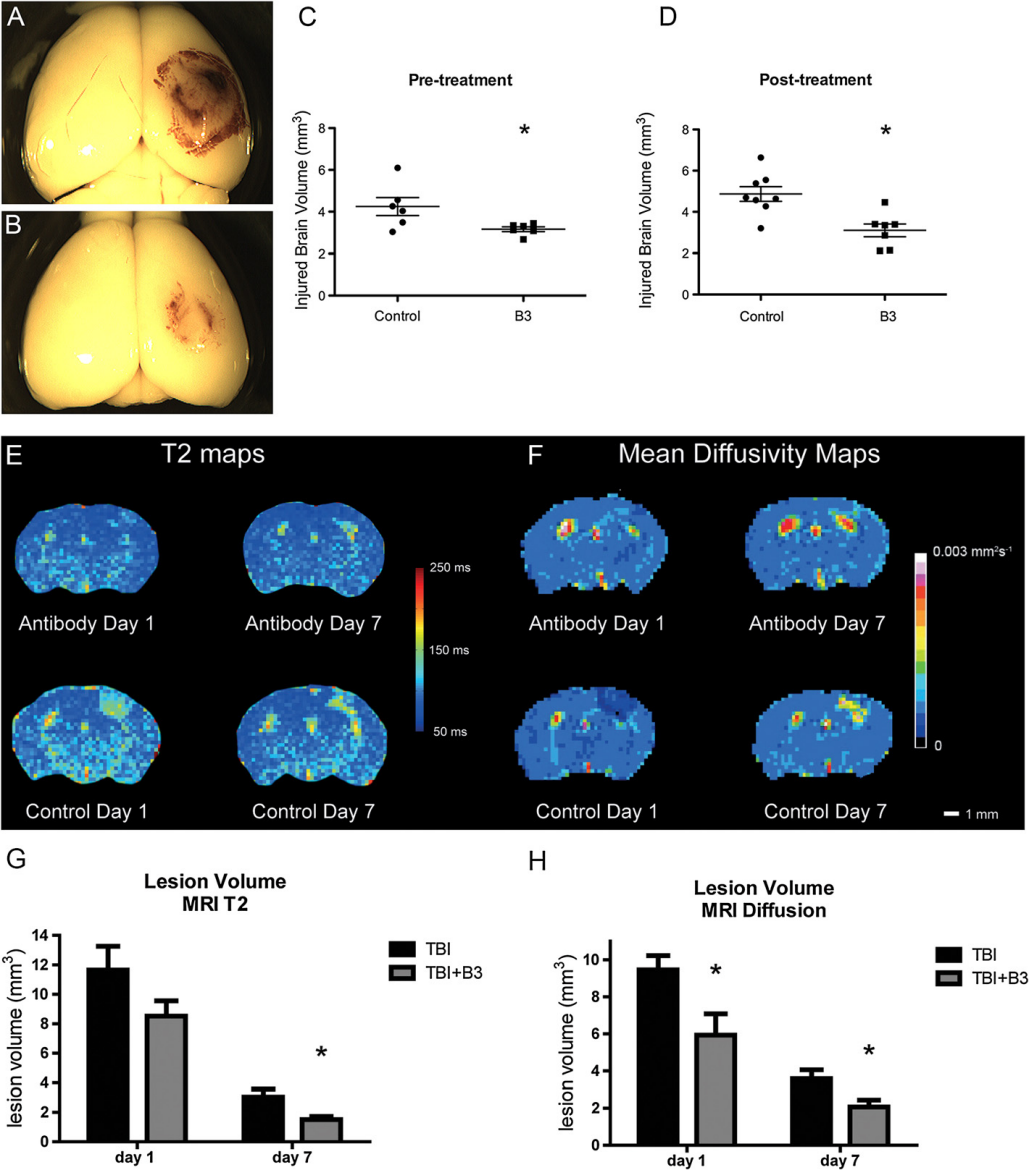
**B3 significantly reduces lesion volume and diffusion following traumatic brain injury (TBI). (A, B)** Representative images of mouse brains 2 days following TBI, isotype control **(A)** or B3 **(B)** received 30 minutes post injury, showing extent of hemorrhage. **(C, D)** Representative quantification of injury volume 2 days following TBI after Nissl 2 staining, with treatment received 1 hour pre-TBI **(C)** or 30 minutes post-TBI **(D)**; means ± standard error of the mean (SEM), n >7 animals; *P <0.05, **P <0.01 by two-tailed *t*-test. **(E)** Representative T_2_ maps of mouse brain 1 and 7 days following TBI in control animals (isotope) or animals treated with B3 (antibody), both given at 25 mg/kg, 30 minutes post injury, by tail-vein injection. **(F)** Representative mean diffusivity map following the same protocol. **(G)** Quantification of infarct volume using T_2_ maps and **(H)** mean diffusivity maps in isotype controls (TBI) and B3-treated animals (TBI + lysophosphatidic acid (LPA) antibody). Data are means ± SEM, n = 8 animals; *P <0.05 by *t*-test.

In our study, mice received B3 or an isotype-matched control mAb (25 mg/kg) in a double-blinded manner given intravenously by tail-vein injection 1 h prior or 30 minutes post TBI and were sacrificed 48 h later, for lesion measurement. As shown in Figure 2, B3 treatment reduced the hemorrhage normally observed following CCI. These data show that compared to isotype control, B3 significantly reduced the subdural hematoma and the lesion area (Figure 2A, B respectively). Compared to controls, brain lesion volume also decreased significantly by 25.5 ± 2.7% (Figure 2C, n = 6) when mice were treated with B3 60 minutes prior to injury. Importantly, a single dose of B3 administered 30 minutes after TBI resulted in a 36.4 ± 6.4% reduction in lesion volume in B3 treatment compared to control (Figure 2D, n ≥7).

MRI scans were also used to assess lesion size and diffusion characteristics over a temporal profile (Figure 2E-H). Animals received a single tail-vein administration of B3 or isotype control 30 minutes after TBI and were then imaged by MRI on days 1 and 7. We observed a reduction of lesion volume in all animals after 7 days when compared to day 1. Importantly, B3 treatment further reduced the lesion volume, with a statistically significant difference observed at 7 days as delineated on the T_2_ maps and at 1 and 7 days as delineated on the mean diffusivity maps. The mean diffusivity maps show a significance decrease in edema in the B3-treated group at both day 1 and day 7 compared to mice receiving isotype-matched mAb control. This finding is mirrored in the T_2_ maps with a trend at day 1 and significance seen at day 7. Together, these data demonstrate that anti-LPA treatment reduces the lesion size of TBI and accompanying edema.

### Anti-LPA treatment improves behavioral deficits after TBI

**Figure 3:**
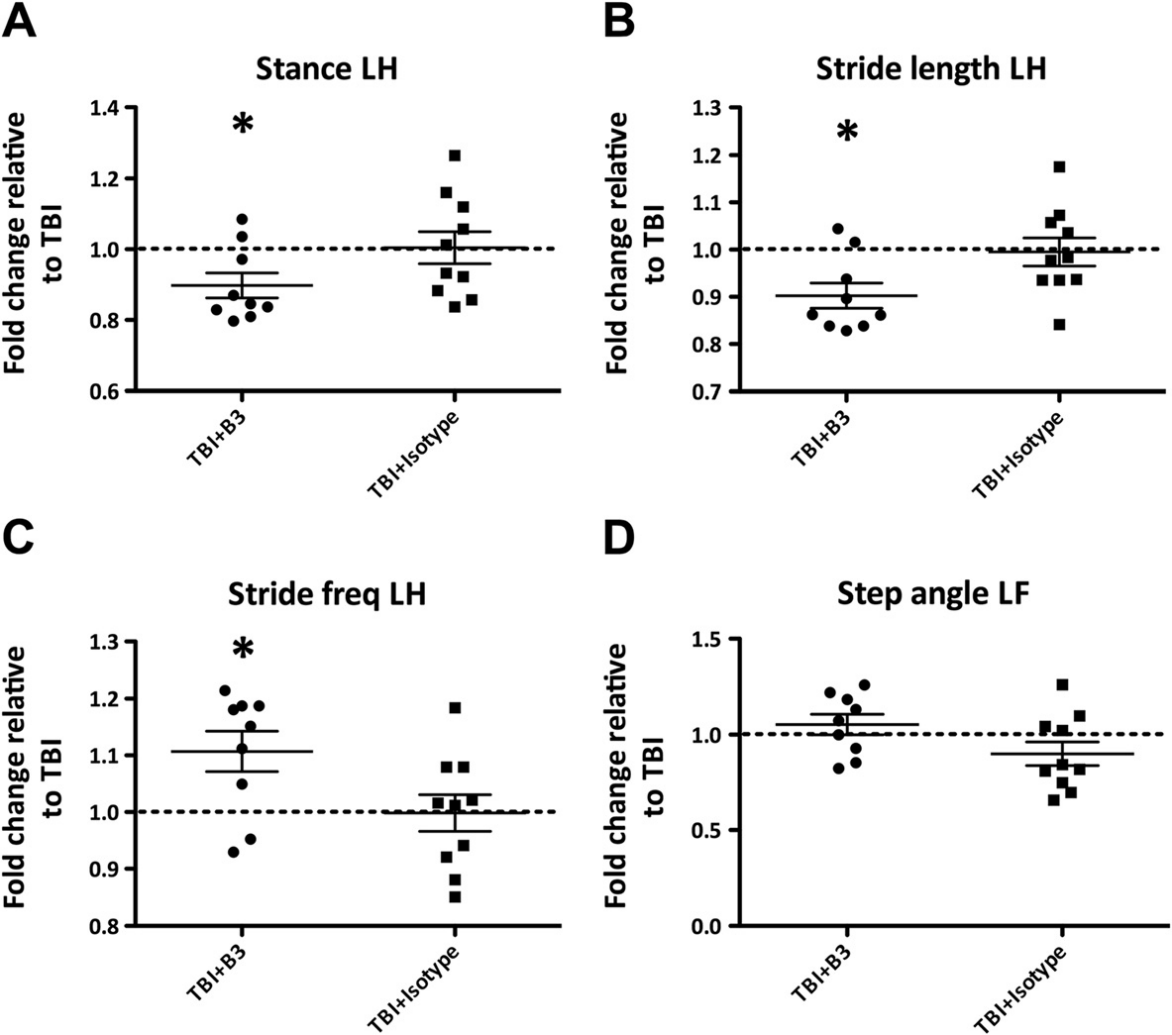
**B3 treatment post TBI significantly improves behavioral outcome.** At 48 h after traumatic brain injury (TBI), mice treated with B3 display improvement in the left hind limb parameters of stance **(A)**, stride length **(B)**, stride frequency **(C)** and step angle **(D)**. B3 and isotype control values are presented as fold-change to TBI. Data represent mean ± standard error of the mean (SEM), *P <0.05, n = 10 animals per group.

The DigiGait system and software were used to assess changes in neurological function in mice after TBI. We established that injured mice displayed impairments in motor functions in their left hind and fore limbs (contralateral limbs to brain injury side) compared to sham mice in parameters such as stance/swing ratio, % swing in stride, stance and stride frequency (data not shown). All gait indices calculated for the B3- and isotype control-treated mice are presented as fold-change to TBI. Importantly, the administration of B3, 30 minutes post TBI significantly improved behavioral outcomes of the parameters of stance, stride length and stride frequency (Figure 3A-C) and there was a trend to significance in step angle, however this was not statistically significant (Figure 3D). These results indicate that B3 treatment post TBI led to improvements in neurological function after TBI. This long-term behavioral benefit in addition to the acute improvements in injured brain volume seen in Figure 2, suggests that anti-LPA antibodies have neuroprotective properties in attenuating both the immediate and secondary phases of brain injury.

### Anti-LPA treatment decreases IL-6 after TBI

**Figure 4:**
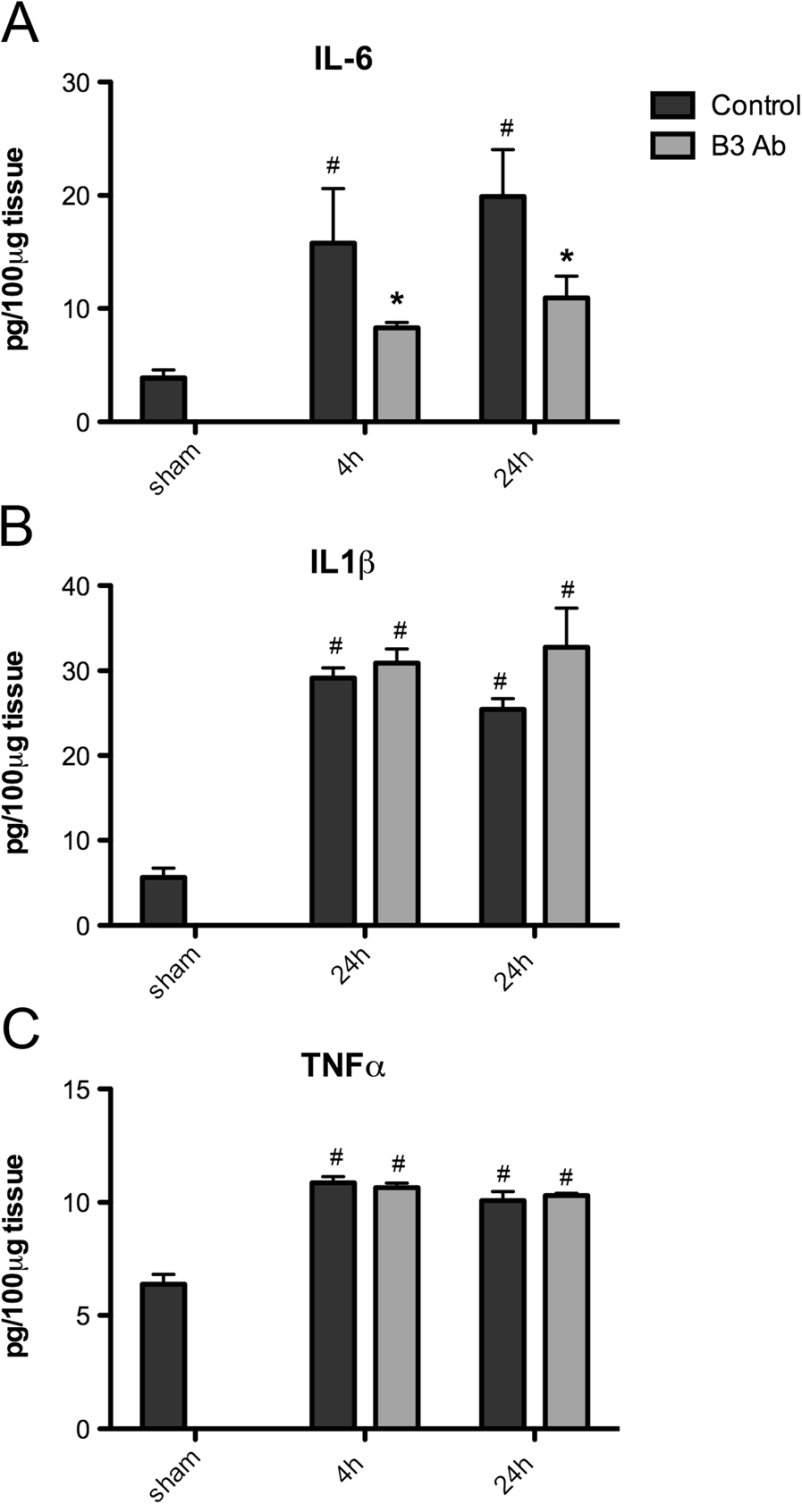
**B3 significantly reduces IL-6 production comparable with sham levels but not IL1-β and TNF-α following traumatic brain injury (TBI).** The graphs show the levels of cytokines in the ipsilateral hemisphere of mouse brains at 4 and 24 h following TBI. **(A)** Il-6. **(B)** IL-1β **(C)** TNF-α. Animals received a single intravenous injection of B3 (25 mg/kg) or its isotype control (Ab isotype), 30 minutes post injury. Data are mean ± standard error of the mean of triplicates. Statistical significance of B3 treatment was detected by one way analysis of variance followed by Bonferroni post hoc analysis, *P <0.05; significance of cytokine levels as a consequence of TBI injury is indicated by ^#^*P* <0.05.

We assessed whether or not anti-LPA mAb treatment could have an anti-inflammatory action by examining the expression levels of key inflammatory cytokine known to contribute to the secondary phases of neurotrauma. For example, IL-6 and IL-1β are important cytokines with levels that correlate with poor outcomes and behavioral defects, and it has been suggested that preventing IL-6 and IL-1β upregulation could have therapeutic benefit in TBI [14]. As expected in our model of CCI we observed a rapid (4 h) upregulation of IL-6 protein concentration following TBI, in both hemispheres (Figure 4). Interestingly, the ELISA data showed a statistically significant decrease in IL-6 protein levels after treatment with B3 at both 4 and 24 h after TBI when compared to the isotype control (Figure 4A). Importantly, levels of IL-6 observed in the presence of B3 at both time points were not statistically different from the levels observed in sham (Figure 4A). However, unlike IL-6, the levels of IL-1β and TNFα were not altered after B3 treatment. The dramatic effect of anti-LPA mAb treatment in reducing IL-6 production suggests that the structural and functional improvement achieved with antibody treatment involved LPA-mediated induction of IL-6. Moreover, the data corroborate a role of LPA in enhancing the inflammatory process that follows TBI and raises the possibility that LPA might modulate the production of key inflammatory cytokines, such as IL-6, following neurotrauma.

## Discussion

Currently, there are no Food and Drug Administration (FDA)-approved drugs specifically developed for the treatment of TBI. The high frequency of morbidity and mortality in these patients is attributed to the lack of therapeutic agents that address the mechanisms involved in brain injury. Here, we provide evidence for a role of the pro-inflammatory bioactive lipid LPA, in contributing to TBI neurotrauma by demonstrating the efficacy of our anti-LPA mAbs to mitigate early effects of injury such as hemorrhage, edema and injured brain volume as well as mitigating the longer-term behavioral deficits.

It is likely that the reduced secondary effects mediated by anti-LPA treatment are due to the ability of the antibody to block LPA-induced release of inflammatory cytokines, particularly IL-6. Upregulation of IL-6 in the brain is considered to be a main driver of the inflammatory response after TBI [15] and contributes to the behavioral deficits, as demonstrated by the ability of anti-IL-6 antibody treatment to mitigate brain injury and behavioral responses in the mouse model of TBI and hypoxia [15]. This is consistent with the association between elevated serum levels of IL-6 and poor outcomes in TBI patients [16,17].

Importantly, our study is also the first to demonstrate that the LPA levels are dysregulated in the CSF as a consequence of brain injury in humans as well as in mice. It appears that the LPA-pulse occurs in the early, primary phase of brain injury, as we observed a marked increase as early as 4 h post injury in the mouse CCI model and within 24 h in TBI patients. This suggests that LPA may be one of the first players in response to injury and that it could contribute to both primary and secondary sequelae of events. Presumably, the LPA-pulse was modified by our anti-LPA mAb treatment such that the neurotoxic and pro-inflammatory effects of LPA on brain IL-6 levels were reduced. Both CSF and plasma are known to contain one of the major enzymes involved in LPA production, autotaxin, as well as its lysophospholipid substrates [18-20]. The beneficial effects of our antibody therapy in reducing hemorrhage and edema can be explained by the ability of LPA to enhance the permeability of the blood brain barrier (BBB) in vivo [21]. It is thus possible that the dysregulated LPA observed following TBI contributes to an increase permeability of the BBB, an effect that could thus potentially be blocked by anti-LPA mAb treatment.

Our most compelling evidence for a causative role of LPA in murine TBI comes from studies using a specific murine mAb against LPA, B3. A single intravenous administration given 30 minutes after injury to CCI mice was able to significantly and substantially reduce the lesion size as assessed both histologically and by T_2_-weighted MRI. This neuroimaging technique is employed routinely in clinical settings to characterize the extent of brain injury and measure ensuing edema.

The observed near-term neuroprotective effects of the anti-LPA mAb can be explained by the well-known effects of LPA in inducing necrosis in cultured neurons, promoting neurite retraction and preventing neuronal differentiation and development (reviewed in [2]), effects that are, in part, reversed by antibody treatment. These neuroprotective actions seen in the TBI model corroborate our previous studies showing upregulation of LPA receptors in the injured mouse spinal cord and human brain [3,4] and that blocking LPA signaling with B3 improves outcome in mice subjected to SCI [5].

The potent efficacy of the anti-LPA mAb in mitigating trauma in the murine CCI model, together with the fact that LPA levels were also dysregulated in the mouse CSF following TBI, indicate that the findings from this model may translate well into human TBI therapies and that our humanized variant of the anti-LPA mAb could be a novel treatment for the condition. Neutralizing extracellular LPA signaling could be the first therapeutic agent to mitigate both primary and secondary phases of neurotrauma, with resulting potential beneficial outcomes in rehabilitation and functional recovery for the patients. A variety of therapeutic interventions in the LPA signaling pathway could be envisioned and have been discussed previously [2]. One could develop small molecule inhibitors targeting either the LPA receptors or key enzymes in the biosynthetic machinery responsible for LPA production. Regarding LPA receptor antagonists, there is the problem that there are six G protein-coupled receptors (GPCRs) for LPA plus two purinergic receptors and possibly one ion channel (transient receptor potential cation channel subfamily V member 1 (TRPV1)) This receptor redundancy may require the development of a pan-LPA receptor antagonist, as there are several LPA receptors expressed in the human brain after neurotrauma [3]. This would pose a hurdle for medicinal chemistry. Alternately, one could target the upstream biosynthetic machinery responsible for LPA upregulation in the CNS. Although autotaxin (ATX) is a major source of LPA in the blood and some tissue, it may not be the source of dysregulated LPA after neurotrauma and future work will be needed to determine the role of LPA production including phospholipase (PLA)_1_, PLA_2_, alpha glycerol kinase (AGK) and other monoacylglycerol (MAG)-kinases as well as glycerol-3-phosphate acyltransferase (GPAT). Lysophosphatidyl choline (LPC), the substrate for LPA production by ATX, is very low in human CSF even after TBI (50 nM) and is consistent with previous reports of low LPC in normal CSF [22], and we would argue that ATX may not be the key enzyme responsible for LPA production. However, further experiments are planned to determine the source of the LPA.

It is interesting to note that the LPA profile (Figure 1) suggests that the brain produces a pattern of LPA species quite distinct from plasma. LPA 18:0 is overwhelmingly the predominant species in the injured mouse CSF. The 18:0 is also a major LPA isoform in the injured human CSF but 16:0 also appears to be predominant. It is interesting to note that these species are not the most abundant in plasma. This needs to be confirmed in more detailed studies that are planned, but suggests at least, that hemorrhage (that is, blood source) is not likely the main source of LPA after TBI. One could further argue against the blood source of the LPA-pulse in that the concentration of LPA in CSF (approximately 2 μM for mouse) is much higher than the 50 to 100 nM levels typically observed in plasma [23]. One would suspect that the plasma source of LPA would be diluted even more when mixed with the CSF. An additional argument against plasma as the source of the LPA-pulse is the finding that LPC levels are in the range of 50 nM for CSF (and do not change after TBI), while LPC levels are in the 50 to 100 microM range in the plasma [23], indicating that the ratio of LPA/LPC is quite different between plasma and CSF, including after TBI.

In vitro studies using the Kinetic Exclusion Assay (KinExA) [24] (Table 2) demonstrate that the anti-LPA mAbs recognize all relevant LPA species, particularly the unusual 18:0 and 16:0 LPAs that are abundant in the CSF of injured mice and humans.

**Table 2:** **KinExA measurements of the apparent equilibrium dissociation constants (K**_**D, app**_**) for B3 binding individual LPA species**

**LPA species**	**K**_**D, app **_**(nM)**	**95% CI (nM)**
16:0	0.88	0.65, 1.1
18:0	14	12, 16
18:1	3.1	2.6, 3.5
18:2	1.5	1.3, 1.7
20:4	5.7	5.3, 6.0

KinExA data were analyzed using the standard analysis method with drift correction. All analysis carried out using KinExA Pro software.

The apparent affinities of the B3 mAb for the various LPA species are at a minimum of four times higher but typically two to three orders of magnitude higher than the affinities enjoyed by the LPA receptors as reported in the literature, affinities reported to be between approximately 60 and 800 nM [25,26]. This is a complicated issue as each receptor has different affinities for the various LPAs, as they are not all equally potent in activating a particular receptor. Despite this, our antibodies are potent in vitro with regard to binding up all relevant LPA species. Moreover, one might argue that the important proof of potency is that our antibodies demonstrate in vivo efficacy reported here and in mitigating SCI [5].

It could be argued that the use of a LPA-neutralizing antibody could provide an alternate approach to reducing upregulated LPA signaling. Neutralizing extracellular LPA with an antibody would silence all of the LPA receptors and ion channel targets, and would not discriminate on LPA synthesized by a variety of enzymatic sources, The disadvantages of antibody therapeutics is the high cost of the drug product, plus the drug must have access to the tissue compartment where the target (that is, LPA) is dysregulated. Fortunately for antibody therapeutics in the TBI context, the BBB of the TBI patient is usually permeable to large proteins like therapeutic antibodies during the critical-care period where salvaging of damaged nerve tissue would be attempted.

We have recently humanized and affinity-matured the anti-LPA mAb by grafting the murine CDR regions on to a human IgGκ1 framework. This preclinical drug candidate, LT3114, retains the specificity for key LPA isoforms, plus it exhibits high affinity enjoyed by the murine B3 mAb. The humanized antibody could serve as a potential therapeutic agent for neurotrauma by limiting the initial injury and, at the same time, reducing dangerous inflammatory processes, possibly closing down the permeabilized BBB while stimulating regenerative processes. Thus, neutralizing extracellular LPA with the humanized mAbs could be the first therapeutic agent that mitigates both early and late phases of neurotrauma with resulting potential beneficial outcomes in rehabilitation and functional recovery for patients.

## Abbreviations

AGK: Alpha glycerol kinase; ATX: Autotaxin; BBB: Blood brain barrier; BSA: Bovine serum albumin; CCI: Control cortical impact; CNS: Central nervous system; CSF: Cerebrospinal fluid; ELISA: Enzyme-linked immunosorbent assay; EVD: Extraventricular drain device; FAF-BSA: Fatty acid-free bovine serum albumin; GOSE: Extended Glasgow outcome scale; GPAT: Glycerol-3-phosphate acyltransferase; GCS: Glasgow coma scale; H&E: Hematoxylin and eosin; ICP: Intracranial pressure; IL: Interleukin; ISF: Inverse of the sampling fraction; ISS: Injury severity score; LPA: Lysophosphatidic acid; LPC: Lysophosphatidyl choline; mAb: Monoclonal antibody; MRI: Magnetic resonance imaging; MRM: Multiple reaction monitoring; PBS: Phosphate-buffered saline; PLA: Phospholipase; RARE: Rapid acquisition, relaxation enhanced; SCI: Spinal cord injury; TBI: Traumatic brain injury; TNF: Tumor necrosis factor.

## Competing interests

RAS, JKF and JMW have stock in Lpath.

## Authors’ contributions

PJC, RAS and AP conceived the experiments. MZ, IK, MS, RLM, and MD performed TBI experiments and behavioral analysis. YG performed immunochemistry. MZ, DW and LAJ performed MRI experiments. RAS developed the anti-LPA mAbs. ACMK and AC collected CSF samples. AM and JMW performed HPLC MS measurements. JKF and JMW performed the KinExA assays. All authors contributed to data analysis and writing of the manuscript. PJC, RAS, and AP contributed financial support for this work. All authors have read and approved the final version of the manuscript.

## References

[B1] Rosenfeld JV Maas AI Bragge P Morganti-Kossmann MC Manley GT Gruen RL Early management of severe traumatic brain injury Lancet 2012 380 1088 1098 10.1016/S0140-6736(12)60864-2 22998718

[B2] Frisca F Sabbadini RA Goldshmit Y Pebay A Biological effects of lysophosphatidic acid in the nervous system Int Rev Cell Mol Biol 2012 296 273 322 22559941 10.1016/B978-0-12-394307-1.00005-9

[B3] Frugier T Crombie D Conquest A Tjhong F Taylor C Kulkarni T McLean C Pebay A Modulation of LPA receptor expression in the human brain following neurotrauma Cell Mol Neurobiol 2011 31 569 577 10.1007/s10571-011-9650-0 21234797 PMC11498475

[B4] Goldshmit Y Munro K Leong SY Pebay A Turnley AM LPA receptor expression in the central nervous system in health and following injury Cell Tissue Res 2010 341 23 32 10.1007/s00441-010-0977-5 20495828

[B5] Goldshmit Y Matteo R Sztal T Ellett F Frisca F Moreno K Crombie D Lieschke GJ Currie PD Sabbadini RA Pebay A Blockage of lysophosphatidic acid signaling improves spinal cord injury outcomes Am J Pathol 2012 181 978 992 10.1016/j.ajpath.2012.06.007 22819724 PMC3432439

[B6] Seifman MA Adamides AA Nguyen PN Vallance SA Cooper DJ Kossmann T Rosenfeld JV Morganti-Kossmann MC Endogenous melatonin increases in cerebrospinal fluid of patients after severe traumatic brain injury and correlates with oxidative stress and metabolic disarray J Cereb Blood Flow Metab 2008 28 684 696 10.1038/sj.jcbfm.9600603 18183032

[B7] Chi JH Knudson MM Vassar MJ McCarthy MC Shapiro MB Mallet S Holcroft JJ Moncrief H Noble J Wisner D Kaups KL Bennick LD Manley GT Prehospital hypoxia affects outcome in patients with traumatic brain injury: a prospective multicenter study J Trauma 2006 61 1134 1141 10.1097/01.ta.0000196644.64653.d8 17099519

[B8] Marshall LF Marshall SB Klauber MR Van Berkum CM Eisenberg H Jane JA Luerssen TG Marmarou A Foulkes MA The diagnosis of head injury requires a classification based on computed axial tomography J Neurotrauma 1992 9 Suppl 1 S287 S292 1588618

[B9] Baker SP O’Neill B Haddon W Jr Long WB The injury severity score: a method for describing patients with multiple injuries and evaluating emergency care J Trauma 1974 14 187 196 10.1097/00005373-197403000-00001 4814394

[B10] Federico L Ren H Mueller PA Wu T Liu S Popovic J Blalock EM Sunkara M Ovaa H Albers HM Mills GB Morris AJ Smyth SS Autotaxin and its product lysophosphatidic acid suppress brown adipose differentiation and promote diet-induced obesity in mice Mol Endocrinol 2012 26 786 797 10.1210/me.2011-1229 22474126 PMC3355557

[B11] Dixon CE Clifton GL Lighthall JW Yaghmai AA Hayes RL A controlled cortical impact model of traumatic brain injury in the rat J Neurosci Methods 1991 39 253 262 10.1016/0165-0270(91)90104-8 1787745

[B12] Sashindranath M Sales E Daglas M Freeman R Samson AL Cops EJ Beckham S Galle A McLean C Morganti-Kossmann C Rosenfeld JV Madani R Vassalli JD Su EJ Lawrence DA Medcalf RL The tissue-type plasminogen activator-plasminogen activator inhibitor 1 complex promotes neurovascular injury in brain trauma: evidence from mice and humans Brain 2012 135 3251 3264 10.1093/brain/aws178 22822039 PMC3501968

[B13] Sashindranath M Samson AL Downes CE Crack PJ Lawrence AJ Li QX Ng AQ Jones NC Farrugia JJ Abdella E Vassalli JD Madani R Medcalf RL Compartment- and context-specific changes in tissue-type plasminogen activator (tPA) activity following brain injury and pharmacological stimulation Lab Invest 2011 91 1079 1091 10.1038/labinvest.2011.67 21519332

[B14] Rasouli J Lekhraj R White NM Flamm ES Pilla AA Strauch B Casper D Attenuation of interleukin-1beta by pulsed electromagnetic fields after traumatic brain injury Neurosci Lett 2012 519 4 8 10.1016/j.neulet.2012.03.089 22503903

[B15] Yang SH Gangidine M Pritts TA Goodman MD Lentsch AB Interleukin 6 mediates neuroinflammation and motor coordination deficits after mild traumatic brain injury and brief hypoxia in mice Shock 2013 40 471 475 10.1097/SHK.0000000000000037 24088994 PMC4218737

[B16] Minambres E Cemborain A Sanchez-Velasco P Gandarillas M Diaz-Reganon G Sanchez-Gonzalez U Leyva-Cobian F Correlation between transcranial interleukin-6 gradient and outcome in patients with acute brain injury Critical Care Med 2003 31 933 938 10.1097/01.CCM.0000055370.66389.59 12627008

[B17] Hergenroeder GW Moore AN McCoy JP Jr Samsel L Ward NH 3rd Clifton GL Dash PK Serum IL-6: a candidate biomarker for intracranial pressure elevation following isolated traumatic brain injury J Neuroinflam 2010 7 19 10.1186/1742-2094-7-19 PMC2853529 20222971

[B18] Koike S Keino-Masu K Ohto T Masu M The N-terminal hydrophobic sequence of autotaxin (ENPP2) functions as a signal peptide Genes Cells 2006 11 133 142 10.1111/j.1365-2443.2006.00924.x 16436050

[B19] Boutin JA Ferry G Autotaxin Cell Mol Life Sci 2009 66 3009 3021 10.1007/s00018-009-0056-9 19506801 PMC11115523

[B20] van Meeteren LA Ruurs P Stortelers C Bouwman P van Rooijen MA Pradere JP Pettit TR Wakelam MJ Saulnier-Blache JS Mummery CL Mummery CL Moolenaar WH Jonkers J Autotaxin, a secreted lysophospholipase D, is essential for blood vessel formation during development Mol Cell Biol 2006 26 5015 5022 10.1128/MCB.02419-05 16782887 PMC1489177

[B21] On NH Savant S Toews M Miller DW Rapid and reversible enhancement of blood–brain barrier permeability using lysophosphatidic acid J Cereb Blood Flow Metabol 1944–1954 2013 33 10.1038/jcbfm.2013.154 PMC3851904 24045401

[B22] Mandal R Guo AC Chaudhary KK Liu P Yallou FS Dong E Aziat F Wishart DS Multi-platform characterization of the human cerebrospinal fluid metabolome: a comprehensive and quantitative update Genome Med 2012 4 38 10.1186/gm337 22546835 PMC3446266

[B23] Nakamura K Kishimoto T Ohkawa R Okubo S Tozuka M Yokota H Ikeda H Ohshima N Mizuno K Yatomi Y Suppression of lysophosphatidic acid and lysophosphatidylcholine formation in the plasma in vitro: proposal of a plasma sample preparation method for laboratory testing of these lipids Analyt Biochem 2007 367 20 27 10.1016/j.ab.2007.05.004 17568554

[B24] Darling RJ Brault PA Kinetic exclusion assay technology: characterization of molecular interactions Assay Drug Dev Technol 2004 2 647 657 10.1089/adt.2004.2.647 15674023

[B25] Fujiwara Y Sardar V Tokumura A Baker D Murakami-Murofushi K Parrill A Tigyi G Identification of residues responsible for ligand recognition and regioisomeric selectivity of lysophosphatidic acid receptors expressed in mammalian cells J Biol Chem 2005 280 35038 35050 10.1074/jbc.M504351200 16115890

[B26] Yanagida K Masago K Nakanishi H Kihara Y Hamano F Tajima Y Taguchi R Shimizu T Ishii S Identification and characterization of a novel lysophosphatidic acid receptor, p2y5/LPA6 J Biolog Chem 2009 284 17731 17741 10.1074/jbc.M808506200 PMC2719412 19386608

